# Histological Correlates of Diffusion-Weighted Magnetic Resonance Microscopy in a Mouse Model of Mesial Temporal Lobe Epilepsy

**DOI:** 10.3389/fnins.2020.00543

**Published:** 2020-06-03

**Authors:** Katharina Göbel-Guéniot, Johannes Gerlach, Robert Kamberger, Jochen Leupold, Dominik von Elverfeldt, Jürgen Hennig, Jan G. Korvink, Carola A. Haas, Pierre LeVan

**Affiliations:** ^1^Department of Radiology, Medical Physics, Medical Center – University of Freiburg, Freiburg, Germany; ^2^Faculty of Medicine, University of Freiburg, Freiburg, Germany; ^3^Experimental Epilepsy Research, Department of Neurosurgery, Medical Center – University of Freiburg, Freiburg, Germany; ^4^Department of Microsystems Engineering, Technical Faculty, University of Freiburg, Freiburg, Germany; ^5^BrainLinks-BrainTools Cluster of Excellence, University of Freiburg, Freiburg, Germany; ^6^Center for Basics in NeuroModulation (NeuroModulBasics), Faculty of Medicine, University of Freiburg, Freiburg, Germany; ^7^Institute of Microstructure Technology, Karlsruhe Institute of Technology, Karlsruhe, Germany; ^8^Department of Radiology and Department of Paediatrics, Cumming School of Medicine, University of Calgary, Calgary, AB, Canada; ^9^Hotchkiss Brain Institute and Alberta Children’s Hospital Research Institute, University of Calgary, Calgary, AB, Canada

**Keywords:** MR microscopy, HARDI, tractography, hippocampus, mesial temporal lobe epilepsy, kainate

## Abstract

Mesial temporal lobe epilepsy (MTLE) is the most common type of focal epilepsy. It is frequently associated with abnormal MRI findings, which are caused by underlying cellular, structural, and chemical changes at the micro-scale. In the current study, it is investigated to which extent these alterations correspond to imaging features detected by high resolution magnetic resonance imaging in the intrahippocampal kainate mouse model of MTLE. Fixed hippocampal and whole-brain sections of mouse brain tissue from nine animals under physiological and chronically epileptic conditions were examined using structural and diffusion-weighted MRI. Microstructural details were investigated based on a direct comparison with immunohistochemical analyses of the same specimen. Within the hippocampal formation, diffusion streamlines could be visualized corresponding to dendrites of CA1 pyramidal cells and granule cells, as well as mossy fibers and Schaffer collaterals. Statistically significant changes in diffusivities, fractional anisotropy, and diffusion orientations could be detected in tissue samples from chronically epileptic animals compared to healthy controls, corresponding to microstructural alterations (degeneration of pyramidal cells, dispersion of the granule cell layer, and sprouting of mossy fibers). The diffusion parameters were significantly correlated with histologically determined cell densities. These findings demonstrate that high-resolution diffusion-weighted MRI can resolve subtle microstructural changes in epileptic hippocampal tissue corresponding to histopathological features in MTLE.

## Introduction

Epilepsy is a neurological disorder characterized by epileptic seizures caused by pathologically hyper-excitable and hyper-synchronized neuronal networks (Fisher et al., 2005; Ono and Galanopoulou, 2012). The most common form of focal epilepsy is mesial temporal lobe epilepsy (MTLE), which involves the hippocampus (HC), from which seizures frequently originate (Engel, 1996). The disease may develop over the course of several years in humans (Engel, 1996), yet classical MTLE can only be identified in human patients by MRI following the complete development of so-called hippocampal sclerosis, which comprises the degeneration of certain neuronal subpopulations (esp. hilar interneurons and cornu ammonis CA1 and CA3 pyramidal cells), dispersion of the granule cell layer (GCL) and changes of the typical hippocampal innervation pattern (e.g., mossy fiber sprouting) (Haas et al., 2002; Thom et al., 2002; Blümcke et al., 2013). These effects are also mimicked by the kainate mouse model of MTLE (Suzuki et al., 1995; Bouilleret et al., 1999; Riban et al., 2002), where the disease manifests weeks following an initial epileptogenic insult. This model is frequently used to study different cellular and molecular aspects of epileptogenesis (Heinrich et al., 2006, 2011; Häussler et al., 2012; Marx et al., 2013; Janz et al., 2017). These various processes underlying the development of epilepsy are in practice not accessible in humans since they are not accompanied by obvious clinical manifestations, yet the early detection of subtle pathological biomarkers may open up novel avenues for disease treatment even before the occurrence of the first seizure.

For this purpose, high-resolution diffusion-weighted imaging (DWI) and tractography may be particularly promising due to its ability to characterize brain tissue microstructure (Pirttilä et al., 2001; Guilfoyle et al., 2003; Immonen et al., 2008; Ong et al., 2008; Ong and Wehrli, 2010; Hansen et al., 2011; Budde and Frank, 2012; Kharatishvili et al., 2014). Properties of water diffusion can notably be used to infer the presence of white matter fiber bundles, but also reflect the underlying microstructural anatomy in regions with more complex architectures (Shepherd et al., 2006). This has been further confirmed by studies of *ex vivo* samples allowing the direct comparison of diffusion tractography (DT) results with histology of the same tissue (Flint et al., 2010; Hansen et al., 2011).

The hippocampal formation has been of particular interest in MR microscopy studies (Shepherd et al., 2006; Flint et al., 2009; Wu and Zhang, 2016), where it possible to differentiate hippocampal subfields and layers according to their microstructural properties, given sufficient spatial resolutions. Several studies have shown that DWI properties differ between healthy and epileptic hippocampi (Assaf et al., 2003; Liacu et al., 2010; Rutland et al., 2018), but it is not completely clear what the various altered DWI parameters represent with respect to specific cellular-level aspects of epileptogenesis. In this study, we thus investigate the relationship between diffusion MR microscopy and the corresponding histologically determined microstructural characteristics of fixed sections of the epileptic mouse brain.

## Materials and Methods

For this study, 400 μm thick paraformaldehyde (PFA)-fixed sections of the hippocampus (21 sections/5 animals) and whole brain (5 sections/4 animals) from healthy and epileptic animals were examined by MR microscopy. Subsequently, tissue architecture and specific cellular features of the same tissue samples were visualized by histological methods to allow a direct comparison of cytoarchitectural details with the acquired MR data. A previous study had shown that MR diffusion parameters (mean diffusivity and fractional anisotropy) exhibited effect sizes of the order of several standard deviations between epileptic and control animals (Janz et al., 2017), hence a relatively low sample size was sufficient to detect these differences for the ensuing histological correlations.

### Animals

Experiments were carried out with 8–9 weeks old C57BL/6N and transgenic Thy1-eGFP mice, in which enhanced green fluorescent protein (eGFP) is expressed under control of the Thy1 promoter (M-line, C57BL/6 background; Feng et al., 2000). In these mice, approximately 20% of principal neurons are eGFP-positive, wherefore their fixed tissue sections were used to visualize histological details of different brain areas following the MR scans. Mice were kept at room temperature in a 12 h light/dark cycle providing food and water *ad libitum*. All animal procedures were performed in accordance with the guidelines of the European Community’s Council Directive of September 22, 2010 (2010/63/EU) and approved by the regional council (Regierungspräsidium Freiburg) and local animal welfare officer, according to the German animal protection act.

### Experimental Epilepsy

Epileptogenesis was induced by unilateral kainate injection into the right dorsal HC. Mice were anesthetized using a mixture of ketamine (100 mg/kg body weight, i.p.), xylazine (5 mg/kg body weight, i.p.) and atropine (0.1 mg/kg) in 0.9% NaCl (saline) and placed in a stereotaxic frame in the prone position. 50 nl of a 20 mM kainate solution (Sigma-Aldrich, St. Louis, MO, United States) in saline were stereotaxically injected (AP = −2.0, ML = −1.5, DV = −1.9 mm, with bregma as reference) over a period of 60 s, using a micro-pump (CMA/100, Carnegie Medicine, Stockholm, Sweden) operating a 0.5 μl micro-syringe (Hamilton, Bonaduz, Switzerland). Mice were kept under observation for several hours after surgery to confirm behavioral status epilepticus, characterized by mild convulsive movements, chewing, rotations, or immobility, as previously described (Riban et al., 2002). Tissue samples were obtained from animals in the chronic phase of epilepsy (21 days after kainate injection). While many processes that are centrally involved in the pathogenesis of epilepsy (e.g., mossy fiber sprouting) are known to progress further and not reach a final state, most histopathological and electrophysiological hallmarks of MTLE with hippocampal sclerosis (e.g., granule cell dispersion, neuronal cell loss, gliosis, recurrent, spontaneous seizures) are known to have largely developed at that stage (Bouilleret et al., 1999; Suzuki et al., 2005; Heinrich et al., 2006; Pallud et al., 2011; Häussler et al., 2012).

### Sample Preparation

Hippocampal sections were prepared as follows: animals were anesthetized in isoflurane and decapitated. The hippocampus was immediately dissected in ice-cold phosphate buffer (PB), cut into thick transverse sections (400 μm) using a McIlwain Tissue Chopper. Afterward, hippocampal sections were immersion-fixed in 4% PFA over night at 4°C and, subsequently, rinsed 4–5 times, 10 min each, in 5 ml PB.

Brain sections were obtained as follows: mice were transcardially perfused for 1 min with saline, followed by 5 min of perfusion with 4% PFA in 0.1M PB. Afterwards, the brain was removed, post-fixed in 4% PFA (4–6 h at 4°C), rinsed in PB overnight and sectioned on a vibratome (400 μm coronal plane).

Considering the use of multiple sections at different positions along the hippocampal septotemporal axis from the same animals, a total of 21 hippocampal sections (six from control animals as well as nine from the ipsilateral hippocampus and six from the contralateral hippocampus of epileptic animals) and five whole-brain sections (one from a control animal and four from epileptic animals) were investigated.

### MRI Data Acquisition

#### MR Setup

All MR experiments were performed on a horizontal preclinical 7.0 T Bruker BioSpec 70/20 USR system (300 MHz) (BrukerBioSpin, Ettlingen, Germany) with a clear bore diameter of 200 mm and using a Bruker quadrature transmit/receive MRI CryoProbe.

#### Sample Containers

Small MR compatible polymethylmethacrylate (PMMA) containers were glued on a custom-built 3D-printed sample holder and then horizontally mounted on a conventional mouse bed as close as possible to the surface cryoprobe head to minimize signal loss. To avoid dehydration of the tissue, the containers were filled with an isotonic sodium chloride solution (0.9%) and sealed with adhesive tape.

#### Structural MR Imaging

MR protocols were empirically setup to achieve high-resolution imaging (20 μm in-plane) with a signal-to-noise ratio (SNR) of at least 10.

The fixed hippocampal sections were scanned using FLASH (TR = 300 ms, TE = 18 ms, flip angle = 50°, 20 μm × 20 μm × 104 μm resolution, matrix size 650 × 550, FOV 13 mm × 11 mm, four slices, NEX = 32, scan duration 1 h 28 min) and RARE sequences (TR = 2000 ms, TE = 17 ms, RARE factor 6, 20 μm × 20 μm × 104 μm resolution, matrix size 512 × 480, FOV 10.24 mm × 9.6 mm, four slices, NEX = 32, scan duration 1 h 25 min).

The same scan parameters were used for the whole-brain sections, except with a slightly modified field of view and corresponding matrix size in order to avoid foldover artifacts within the imaged tissue: FLASH (matrix size 670 × 500, FOV 13.4 mm × 10 mm, scan duration 1 h 20 min), RARE (matrix size 450 × 750, FOV 9 mm × 15 mm, scan duration 2 h 13 min).

#### Diffusion-Weighted MR Imaging

**FIGURE 1:**
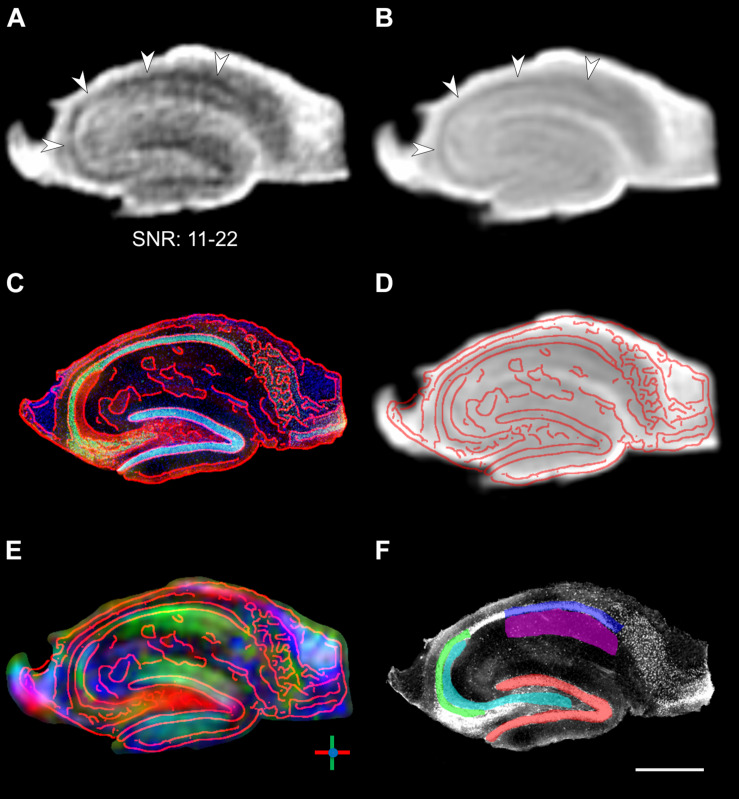
Segmentation of DWI images. **(A)** Raw DWI image of a fixed hippocampal slice with randomly chosen diffusion direction. The b-value of 1000 s/mm^2^ is relatively low for fixed tissue, but nevertheless results in clearly visible signal attenuation in the pyramidal cell layer (arrowheads) and yields acceptable SNR ranging from 11 to 22 across the slice. **(B)** Average DWI images across all 60 directions. **(C)** Corresponding histological image with overlaid contours in red. **(D,E)** Same histological contours overlaid on the mean DWI image and the image of primary diffusion orientations, demonstrating the coregistration of the DWI and histological images. In the diffusion orientation image, dorso-ventral diffusion is shown in green, left–right diffusion is shown in red, and through-plane anterior–posterior streamlines are shown in blue. **(F)** Manual ROI segmentation overlaid on the histological image (same image as in **C**, but displayed in grayscale for clarity): Red: granule cell layer of the dentate gyrus; Cyan: hilus extending into the CA3 stratum lucidum (mossy fibers); Green: CA3 pyramidal cell layer; Blue: CA1 pyramidal cell layer; Magenta: CA1 stratum radiatum (Schaffer collaterals). Scale bar: 500 μm.

High angular resolution diffusion imaging (HARDI) data was acquired using a 2D diffusion-weighted segmented EPI sequence with one b-value at 1000 s/mm^2^ as a compromise between diffusion contrast and SNR. Given the observed mean diffusivities of the order of 0.5 × 10^–3^ mm^2^/s in the investigated fixed tissue (see Figure 5 and Supplementary Table S1), this would result in a theoretical ∼18% loss in contrast compared to a b-value of 2000 s/mm^2^, but also a ∼65% gain in SNR due to diffusion signal attenuation. Since we were mainly interested in in-plane substructures, we also used slices thicker than the in-plane resolution to improve SNR. The resulting raw images exhibited clear contrast in regions of higher diffusivity such as the pyramidal cell layer, while maintaining acceptable SNR > 10 in the diffusion-weighted images to allow for a reliable estimation of diffusion parameters (Figures 1A,B).

For the hippocampal sections the scan parameters were as follows: TR = 3000 ms, TE = 46 ms, 4 segments, 60 directions, 6 b = 0 images, 40 μm × 40 μm × 100 μm resolution, matrix size 320 × 255, FOV 12.8 mm × 10.2 mm, four slices, NEX = 16, scan duration 3 h 31 min.

The same parameters were used for the healthy whole-brain sections, with the exception of a larger field of view and corresponding matrix size to account for the larger sample: matrix size 320 × 400, FOV 12.8 mm × 16 mm. This resulted in a TE of 63 ms.

For the imaging of sections from epileptic mice, the protocol was adapted with more EPI segments to reduce distortions: 6 segments, matrix size 320 × 340, FOV 12.8 mm × 13.6 mm. This resulted in a TE of 56 ms and a total scan duration of 5 h 16 min.

The diffusion images were coregistered to each other using the FSL toolbox (Smith et al., 2004) to compensate for potential image shifts caused by eddy currents. Based on the 60-direction diffusion-weighted data, microstructural pathways were reconstructed by a global tractography algorithm freely available within the Fiber Tool package^1^ running under MATLAB (Reisert et al., 2011). Default parameters were used. Briefly, this method uses simulated annealing optimization to reconstruct a distribution of streamlines within each voxel that best matches the acquired data.

### Histological Examination

Following the MRI measurements, histological details were visualized by three different staining methods: fluorescence immunohistochemistry (IHC), Golgi-Cox impregnation, and DiI-based tracing of neuronal processes. Microscopical analysis was performed with an AxioImager 2 (ZENsoftware; Zeiss, Göttingen, Germany). Images were taken with a digital camera (AxioCam MRm for fluorescence, AxioCam MRc5 for bright field, both Zeiss).

For IHC, sections were cryoprotected in 25% sucrose over night at 4°C, embedded and frozen in Tissue-Tek^TM^ O.C.T Compound (Sakura Finetek Europe B.V., Alphen aan den Rijn, Netherlands) and re-sliced (50 μm) using a Leica CM3050 S cryostat (Leica, Wetzlar, Germany). Cryosections were washed three times, 5 min each in PB and IHC was performed according to standard procedures, using antibodies against neuronal nuclei (NeuN, Merck KGaA, Darmstadt, Germany), neurofilament H (SMI-32, Covance, Inc., Princeton, NJ, United States) and zinc transporter 3 (ZnT3, Synaptic Systems, Göttingen, Germany). Primary antibodies were detected by fluorophore-coupled secondary antibodies (Cy2, Cy3, or Cy5, Jackson ImmunoResearch Laboratories). Sections were counterstained with DAPI and coverslipped with fluorescence mounting medium (Dako, Glostrup, Denmark). Only sections from the center of the initially 400 μm thick tissue samples were used in order to achieve the highest possible comparability with the previously acquired MR data in the same specimen (see below).

Golgi staining was performed on hippocampal sections using the FD Rapid GolgiStain^TM^ Kit. Briefly, after impregnation in solutions containing mercuric chloride, potassium dichromate and potassium chromate, the sections were re-sliced (100–200 μm) on a cryostat, developed, dehydrated and, coverslipped with Hypermount (Thermo Fisher Scientific, Waltham, MA, United States).

For DiI tracing of CA1 pyramidal cells and their axonal inputs from Schaffer collaterals, a solid DiI crystal (diameter approximately 30–50 μm) (Thermo Fisher Scientific, Waltham, MA, United States) was placed into the CA1 stratum radiatum (SR) of a fixed hippocampal section, using a micromanipulator (H. Saur, Reutlingen, Germany). Sufficient diffusion of the dye was achieved by incubation over 1 to 2 weeks in the dark at room temperature, followed by several washing steps in PB and counterstaining with DAPI. Sections were then coverslipped with fluorescence mounting medium (Dako, Glostrup, Denmark) and analyzed.

For quantitative analysis, the images were manually segmented into the following regions of interests (ROIs) using ImageJ software: pyramidal cell layers of CA1 and of CA3, as well as granule cell layer (GCL) of the dentate gyrus (DG). Cell density within each ROI was approximated by the mean intensity of the DAPI channel after background subtraction. The average GCL width was also measured as a marker for epileptogenicity.

### Quantification and Validation of Diffusion Parameters

To allow a direct comparison and validation of the measured MR parameters with histological features, the MR and histological images were realigned to each other by a 2D rigid-body coregistration using the FSL toolbox (Smith et al., 2004), which automatically aligned both datasets using by finding the rigid-body transformation that minimized mutual information as a cross-modality distance measure (Figures 1C–E). The use of tissue samples that were already relatively thinly sliced (400 μm) for the MRI minimized any mismatch in the slice direction. Moreover, while the imaging slices for MRI (100 μm) and the histological slices for IHC (50 μm) had different thicknesses, care was taken to select those slices from the center of the sample for both MRI and IHC, thus ensuring that the histological slice was fully contained within the MRI slice. The following additional ROIs were then defined on the coregistered images: the hilar region extending into the stratum lucidum of CA3 (where we would expect mossy fibers), and the stratum radiatum of CA1 (srCA1, where Schaffer collaterals are expected) (Figure 1F).

Within all ROIs, the fractional anisotropy (FA), mean diffusivity (MD), and primary diffusion orientation were computed from the diffusion tensor fitted to the MR data at each voxel. Additionally, the diffusivity along the dorso-ventral axis (dvD), the diffusivity along the left–right axis (lrD), and the ratio between the two (dvlr_ratio) were computed by fitting a spherical harmonic basis to the acquired HARDI data to interpolate the apparent diffusion coefficient (ADC) profile along the given axes.

Statistical comparisons of the diffusion parameters across ROIs and across groups (control, epileptic ipsilateral, and epileptic contralateral) were performed by ANOVA and *post hoc* Tukey tests. Primary diffusion orientations were tested for uniformity by the V-test. Concordance between the diffusion parameters and the corresponding histological quantities was assessed by Pearson correlation coefficients. In all cases, the statistical significance threshold was set at *p* < 0.05.

## Results

### Imaging of Hippocampal Slices

**FIGURE 2:**
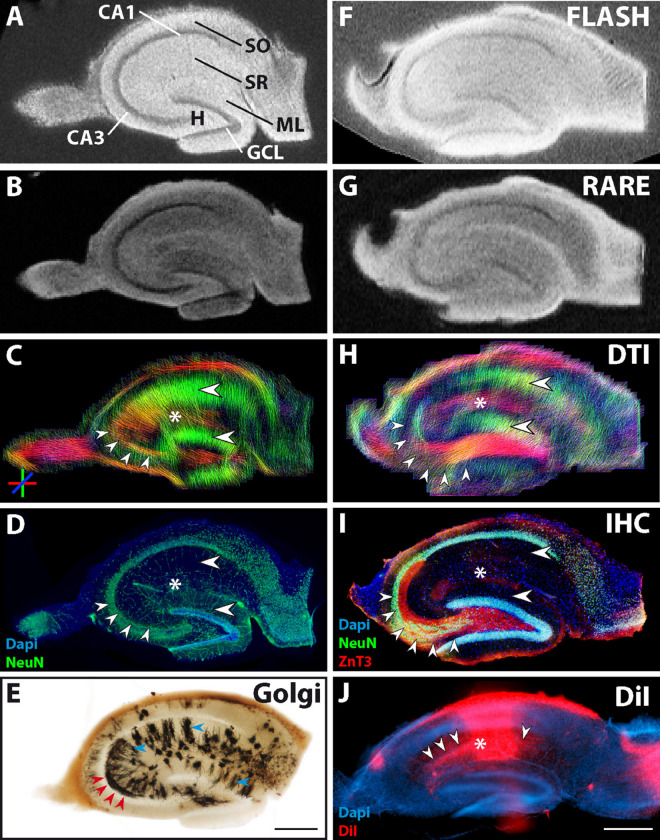
MR and histological images of representative fixed hippocampal sections of two control animals (Section 1: **A–D**, with an adjacent section used for Golgi staining: **E**; Section 2: **F–I**). **(A,B,F,G)** Structural MRI depicts the main neuronal cell layers and tissue architecture [pyramidal cells in cornu ammonis (CA) 1 and 3; granule cell layer, GCL; molecular layer, ML; stratum oriens, SO; str. radiatum, SR; hilus, H]. Comparison of diffusion tractography images **(C,H)** to corresponding NeuN and ZnT3 immunostaining **(D,I)** shows that regions containing parallel extending dendrites of principal neurons evoke corresponding DWI streamlines (large horizontal arrowheads in the ML and CA1 stratum radiatum in **C,H,I**). Furthermore, granule cell axons, extending from the GCL into the CA3 stratum lucidum are clearly visible (small arrowheads in **C,H,I** and red arrowheads in **E**). In turn, axons from CA3 pyramidal cells project to the CA1 stratum radiatum, innervating CA1 pyramidal cell dendrites. These axons (Schaffer collaterals) could also be seen in the DWI images (asterisks in **C,H**) and could be visualized by DiI staining (small arrowheads in **J**). **(E)** Golgi staining of an adjacent section depicts the localization and orientation of principal cell dendrites (blue arrowheads in SR and ML) and parts of mossy fibers in CA3 (red arrowheads). **(J)** DiI crystal placed into the CA1 stratum radiatum, showing the orientation of CA1 pyramidal cells as well as of innervating CA3 Schaffer collaterals (asterisk and arrowheads, respectively). Scale bar: 500 μm.

**FIGURE 3:**
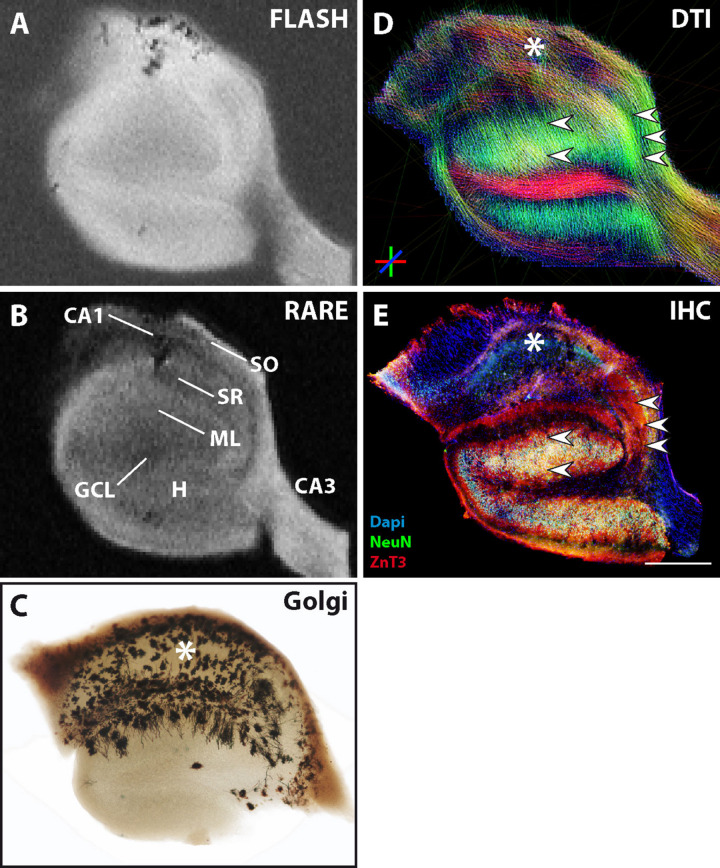
MR and histological images of a representative fixed ipsilateral hippocampal section from a chronically epileptic mouse. **(A,B)** Structural MRI reveals decreased contrast in the pyramidal cell layers compared to control sections (see Figure 2), while the granule cell layer appears thickened. **(E)** Immunostainings showing degeneration of the corresponding neurons (asterisk) and dispersion of the granule cell layer (two arrowheads). **(D,E)** These histopathological changes become even more apparent in DWI, where the loss of CA1 pyramidal cells manifests as a strong reduction in dorso-ventral diffusion streamlines (asterisks in **D,E**). Furthermore, tracts in the granule cell layer appear elongated (two arrowheads in **D**), while the typical mossy fiber streamlines extending from the granule cell layer into the hilus and further into the CA3 region are less pronounced (group of three arrowheads in **D**). These abnormalities correspond to the loss of CA3 target neurons, granule cell layer dispersion and mossy fiber sprouting, which can be seen by NeuN and ZnT3 immunostainings (arrowheads in **E**). **(C)** Golgi staining of an adjacent HC section from a kainate-treated mouse confirms these structural changes and particularly highlights the degeneration and disappearance of CA1 pyramidal cells and their respective dendrites. Instead, many star-shaped glial cells can be seen in the CA1 region (asterisk). Scale bar: 500 μm.

Representative examples of images of control and epileptic slices are shown in Figures 2, 3. In control slices, cell layers of principal neurons were clearly visible in the structural MR images (Figures 2A,B,F,G). Moreover, the diffusion tractography images showed diffusion streamlines with strong directional conformity (Figures 2C,H, big arrowheads) corresponding to radially aligned pyramidal and granule cell dendrites in stratum radiatum and the molecular layer, respectively. These dendritic arborizations could be confirmed by Golgi staining of adjacent sections (Figure 2E, blue arrowheads).

Coherent streamlines were also seen in the stratum oriens, where hippocampal long-range projections are localized, as well as in the stratum lucidum of CA3, into which granule cell axons (mossy fibers) extend, innervating CA3 pyramidal cells. To check potential correspondence with mossy fibers, ZnT3 immunostaining was performed (Figure 2I, red signal, small arrowheads). Parts of the mossy fiber tract could also be found in the Golgi staining (Figure 2E, red arrowheads). Tractography within the region of hilus and CA3 exhibited strong conformity in terms of distribution and directional preference with the corresponding ZnT3 and Golgi staining of mossy fibers (Figures 2C,D,H,I, **small arrowheads and** E, red arrowheads).

DWI also revealed tracts in the stratum radiatum of CA1, which were oriented orthogonally to the previously identified dorso-ventral streamlines of CA1 pyramidal cell dendrites, and likely correspond to CA3 pyramidal cell axons (Schaffer collaterals, Figures 2C,H, color-coded in red and marked by an asterisk). Since these fibers cannot be labeled specifically by antibodies, we placed a DiI crystal into the stratum radiatum of CA1 to stain CA1 pyramidal cells and the innervating Schaffer collaterals (Figure 2J, **asterisk** and **arrowheads**). In the representative photomicrograph shown in Figure 2J, the area of the sections in which the DiI crystal was placed is overexposed due to the high concentration of the dye. Nevertheless, CA1 pyramidal cell dendrites orthogonal to the CA1 pyramidal cell layer can be seen (Figure 2J, asterisk). Additionally, single Schaffer collaterals are stained (Figure 2J, arrowheads), which arise from CA3 pyramidal cells and extend parallel to the CA1 pyramidal cell layer to innervate the CA1 pyramidal cell dendrites.

In epileptic slices, extensive structural changes can already be seen on structural MR images (Figures 3A,B), with strongly reduced contrast in CA1, CA3, and hilar regions along with a dispersed GCL. Double immunostaining for NeuN and ZnT3 revealed the associated neuropathological changes (Figure 3E): a massive thickening of the GCL was apparent (two arrowheads), while CA1 and CA3 pyramidal cells had degenerated (asterisk in CA1). Since CA3 pyramidal cells are target cells of ZnT3-positive mossy fibers, the ZnT3 pattern also changed: some mossy fibers extended to residual CA2 pyramidal cells (Figure 3E, group of three arrowheads), whereas other mossy fibers started to sprout into the dispersed GCL, forming a presumably pro-epileptic positive feedback loop (Figure 3E, two arrowheads). CA2 pyramidal cells were shown to survive after kainate injection and to be innervated by mossy fibers (Häussler et al., 2016). The loss of pyramidal cells could also be seen in the Golgi staining of an adjacent section (Figure 3C, asterisk), where pyramidal cell dendrites are absent. In this region, only a high density of small glial cells was detected.

As in control slices, DWI again provided a more detailed view of these structural features. Streamlines were elongated along the dorsoventral axis in regions of the GCL and molecular layer, most likely originating from granule cell dispersion, mossy fiber sprouting and/or gliosis (Figure 3D, two arrowheads). In the CA2 region, streamlines parallel to the residual CA2 pyramidal cell layer could still be found (Figure 3D, group of three arrowheads). These features could correspond to the portion of mossy fibers which innervate these CA2 cells. In contrast, streamlines were strongly reduced in the stratum radiatum of CA1. Dorsoventral streamlines (green) corresponding to CA1 pyramidal cell dendrites largely disappeared. This reflects the underlying structural state, since pyramidal cells are known to be subject to massive neurodegeneration after kainate injection.

**FIGURE 4:**
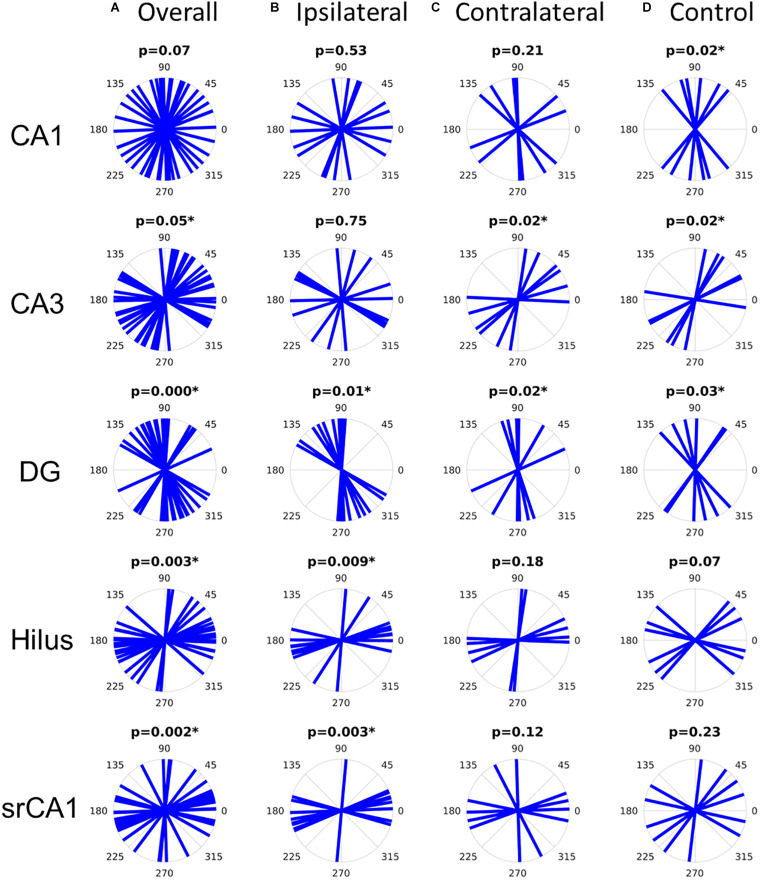
Distribution of primary diffusion orientations in all slices **(A)**, ipsilateral epileptic slices **(B)**, contralateral epileptic slices **(C)**, or control slices **(D)**. The *p*-values refer to the V-test for non-uniformity with hypothesized diffusion orientations with respect to the left–right axis of 90 degrees for CA1 and the dentate gyrus (DG), 0 degrees for the hilus and for the CA1 stratum radiatum (srCA1), and 45 degrees for CA3. Significant *p*-values (asterisks) indicate that the diffusion orientations are not uniformly distributed and rather follow the hypothesized orientations.

The features described above were highly consistent across the studied samples. Primary diffusion orientations were tested against the hypothesis that they mostly lied at specific angles with respect to the left–right axis on the coronal slices: 90 degrees (i.e., dorso-ventral orientation) for diffusion in CA1 and the GCL, corresponding to pyramidal cell and granule cell dendrites, respectively; 0 degrees (i.e., left–right orientation) in the hilus and the stratum radiatum of CA1, corresponding to mossy fibers and Schaffer collaterals, respectively; and 45 degrees in CA3, corresponding to pyramidal cell dendrites. Although it should be noted that these hypothesized orientations were only meant to be approximate, considering that the investigated structures were curved and therefore did not have a truly fixed orientation, the statistical analysis revealed that the measured orientations were concordant with the hypotheses in all cases except in CA1, where the orientations marginally failed to reach significance (*p* = 0.07) (Figure 4A).

However, when dividing the samples into appropriate subgroups (epileptic ipsilateral, epileptic contralateral, and control), it becomes clear that the diffusion orientations in CA1 were actually well-aligned along the dorso-ventral axis in control slices, while they were uniformly distributed in epileptic slices (Figures 4B–D), concordant with the loss of pyramidal cells in the latter. Similarly, in CA3, primary diffusion directions were only consistently oriented in control and contralateral slices, while the loss of pyramidal cells in ipsilateral slices was reflected in a loss of directional conformity (Figure 4B). In the dentate gyrus, all types of slices showed significant directionality along the dorso-ventral axis. In the hilus, only ipsilateral slices showed significant orientation distributions along the left–right axis, consistent with mossy fibers, although control slices only barely failed to reach statistical significance (*p* = 0.07). Finally, in the CA1 stratum radiatum, the loss of pyramidal cell dendrites in ipsilateral slices left Schaffer collaterals as the main source of diffusion anisotropy, thus yielding a statistically significant directional conformity along the left–right axis. This would be consistent with the observation that despite the extensive neuronal cell loss in CA1 and CA3, there is a sprouting response of new Schaffer collaterals between the spared neurons (Siddiqui and Joseph, 2005).

**FIGURE 5:**
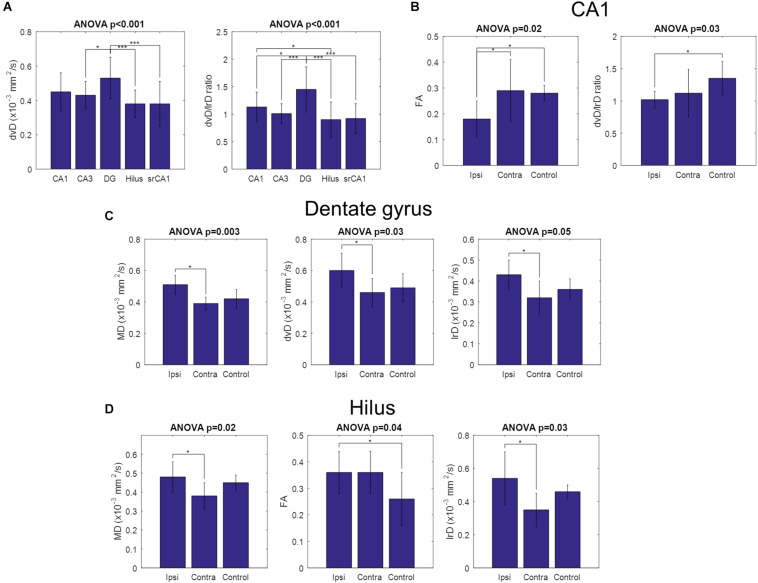
Comparison of diffusion parameters across hippocampal regions and across slice groups. Error bars indicate the standard deviations of the measured parameters. **(A)** Dorso-ventral diffusivity was significantly higher in the dentate gyrus than in CA3, the hilus, and CA1 stratum radiatum. The ratio between dorso-ventral and left–right diffusivities showed a similar pattern, with additional significance between dentate gyrus and CA1, and between CA1 and the hilus. No other diffusion parameters were significantly different across hippocampal regions. **(B)** CA1 group differences. FA was significantly lower in ipsilateral slices than both contralateral and control slices, while the ratio between dorso-ventral and left–right diffusivities was significantly lower in ipsilateral than in control slices. **(C)** Dentate gyrus group differences. Mean diffusivity, dorso-ventral diffusivity, and left–right diffusivity were all significantly higher in ipsilateral than in contralateral slices. **(D)** Hilus group differences. Mean diffusivity and left–right diffusivity were significantly higher in ipsilateral than in contralateral slices, while FA was significantly higher in ipsilateral than in control slices. No other parameters were statistically significant. (*p < 0.05, ***p < 0.001).

As for the other diffusion parameters, they also showed group-level results consistent with the previous observations. The parameters for all regions and groups are shown in Supplementary Table S1. When pooling all slices together, dorso-ventral diffusion was found to be significantly larger in the dentate gyrus than in CA3, the hilus, and srCA1 (Figure 5A). Here, the ratio between dorso-ventral and left–right diffusion was a more sensitive measure, revealing additional statistical significance between dentate gyrus and CA1, and between CA1 and the hilus (Figure 5A). When dividing the slices into appropriate sub-groups, CA1 showed statistically significantly less FA in ipsilateral slices than both contralateral and control slices, as well as statistically significantly less dv/lr ratio in ipsilateral than in control slices (Figure 5B). This again reflects the loss of pyramidal cell dendrites in ipsilateral epileptic slices. In the dentate gyrus, mean diffusion, as well as diffusion along the dorso-ventral and left–right axes, was significantly increased in ipsilateral compared to contralateral slices, possibly reflecting the reduced cell density in the dispersed GCL (Figure 5C). In the hilus, ipsilateral slices had significantly greater mean diffusion and left–right diffusion than contralateral slices (Figure 5D). Ipsilateral slices also had significantly greater FA than control slices, consistent with the emergence of mossy fibers with strong directional conformity in epileptic slices.

**FIGURE 6:**
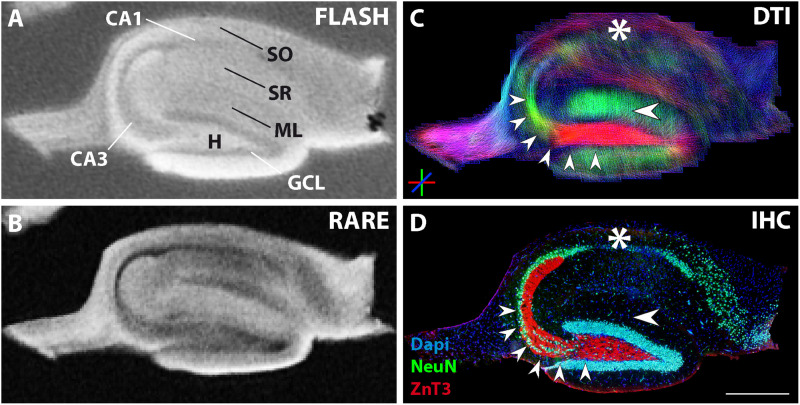
MR and histological images of a representative fixed contralateral hippocampal section from a chronically epileptic mouse with restricted neuronal cell loss in CA1. **(A,B)** Structural MRI shows high contrast in CA3 and the granule cell layer, but diminished contrast in the CA1 pyramidal cell layer. **(C)** The corresponding DWI images show reduced dorso-ventral streamlines within CA1 (asterisk). **(D)** Immuhistochemistry reveals the exclusive degeneration of some but not all CA1 pyramidal cells (asterisk). Other neuronal cell layers or structures like CA3 pyramidal cells, granule cells, and the respective innervation pattern by mossy fibers (small arrowheads) are unaffected. Scale bar: 500 μm.

The previous results showed that contralateral slices sometimes showed similar alterations as ipsilateral slices in comparison to control slices. Indeed, in the kainate mouse model, structural changes may also affect the contralateral hippocampus, depending on the severity of epileptogenesis. One example of such an animal is shown in Figure 6. Immunostaining for NeuN clearly showed that the contralateral side was affected by the kainate injection and that some but not all CA1 pyramidal cells had been lost (Figure 6D, asterisk). In contrast, all the other neuronal cell layers remained unaffected: the layers of CA3 pyramidal cells and granule cells appeared as dense and narrow bands and mossy fibers were clearly extending from the GCL into the CA3 stratum lucidum without any sprouting into the GCL (Figure 6D, small arrowheads). The GCL and CA3 pyramidal layer were visible on structural MRI, but not the CA1 pyramidal layer (Figures 6A,B). In DWI, streamlines corresponding to granule cell dendrites and mossy fibers (Figure 6C, single, horizontal arrowhead and small arrowheads, respectively) precisely matched the underlying cytoarchitecture, but streamlines corresponding to CA1 pyramidal cell dendrites were clearly diminished (Figure 6C, asterisk) and differed from the control situation (Figures 2C,H, **asterisks** and **upper**, big horizontal arrowhead). Therefore, even the impairment of only one neuronal subpopulation without extensive structural changes was clearly visible on MRI.

**FIGURE 7:**
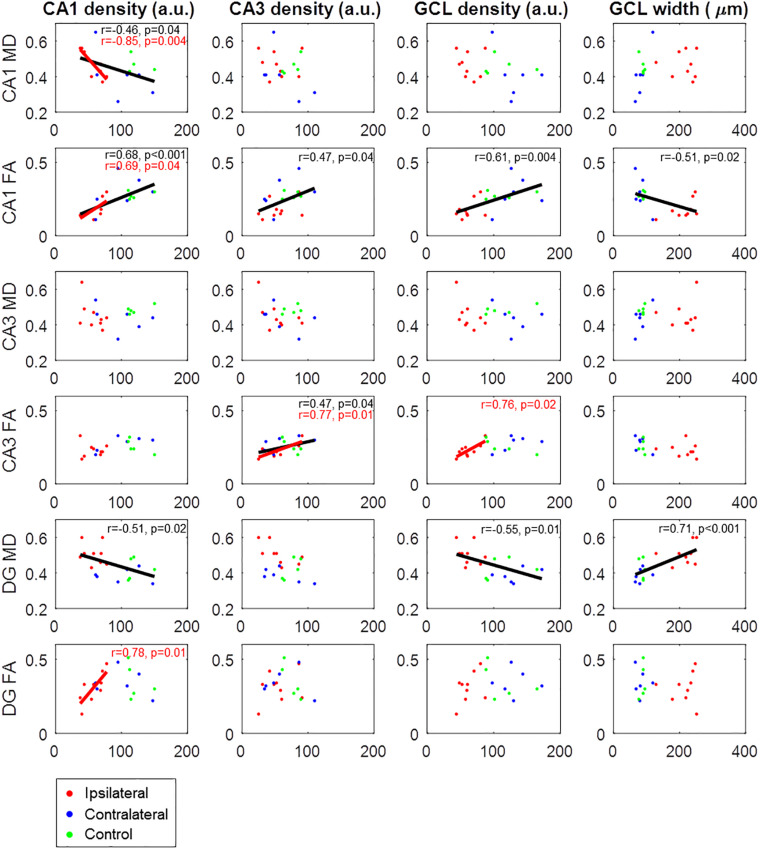
Correlations between histological parameters (columns) and DWI parameters (rows). Cell densities were calculated from the normalized histological image intensities and are thus in arbitrary units (a.u.). Data points are from ipsilateral slices (red), contralateral slices (blue), and control slices (green). Regression lines with Pearson correlation coefficients and corresponding *p*-values are only shown for statistically significant correlations (*p* < 0.05), either across all slices (black) or only considering ipsilateral epileptic slices (red).

A direct comparison of histological and DWI parameters is shown in Figure 7. Significant correlations were observed between corresponding parameters in CA1 (cell density significantly correlated with MD and FA), CA3 (cell density correlated with FA), and the dentate gyrus (cell density correlated with MD). However, such correlations could be merely driven by differences between epileptic and non-epileptic slices. For example, FA of CA1, which we had previously shown to be altered in ipsilateral epileptic slices (see Figure 5), was significantly correlated not only with CA1 cell density, but also with CA3 cell density, GCL density, and GCL width (Figure 7). Similarly, MD of the dentate gyrus was also significantly correlated with multiple histological quantities (Figure 7). As all of these parameters are known to be altered in epilepsy, it is not surprising that significant correlations were observed. Nevertheless, when considering only ipsilateral epileptic slices, significant correlations notably remained between CA1 cell density and both FA and MD of CA1, as well as between CA3 cell density and FA of CA3, thus demonstrating that the DWI parameters directly reflect the underlying cellular microstructure. However, in ipsilateral epileptic slices, GCL density was not significantly correlated with DWI measures in the dentate gyrus, indicating that the diffusion alterations may be driven by features other than granule cells.

### Imaging of Whole-Brain Sections

**FIGURE 8:**
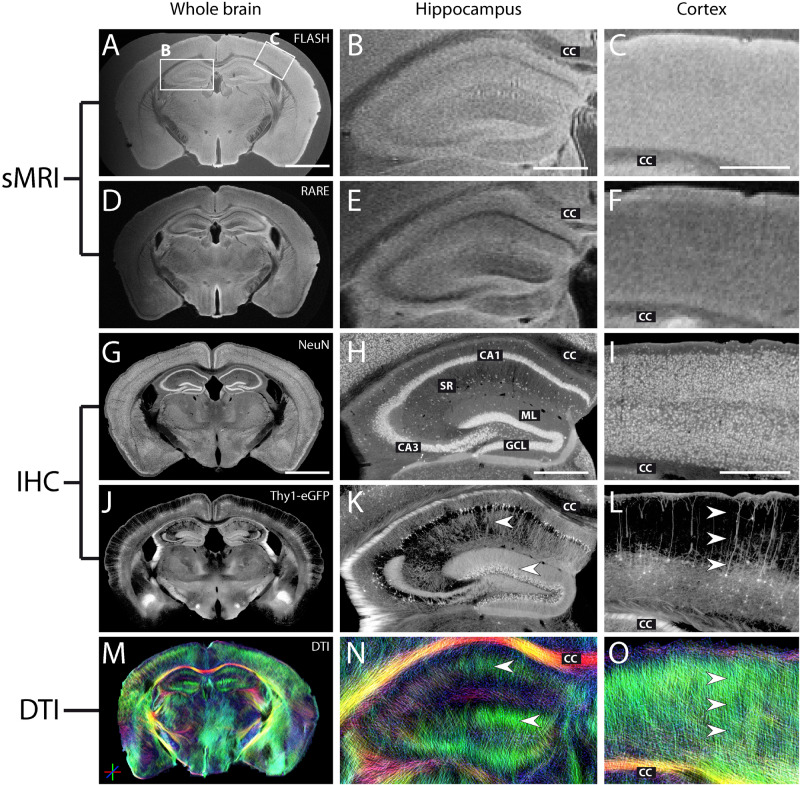
MR and histological images of a representative fixed whole-brain section. **(A–F)** Structural MRI reveals densely packed neuronal cell layers in the hippocampus with a thickness as low as 50 μm, as confirmed by comparison with the corresponding NeuN staining **(G–I)**. Middle and right columns show magnifications of the hippocampus and the neocortex. **(G–I)** NeuN staining highlights regions with high neuronal cell density. While some neurons are arranged in densely packed cell layers (hippocampus, H: pyramidal cells in cornu ammonis CA1-3, GCL: granule cell layer), others can be found in more loosely packed cell layers (cortex, I). **(H,I)** CC, corpus callosum; ML, molecular layer; SR, stratum radiatum. **(J–L)** eGFP expression in single principal neurons illustrates the neuronal cytoarchitecture within different brain regions and highlights major fiber pathways and long-range projections. **(M–O)** DWI shows the well-ordered orientation of neuronal dendritic processes, namely radially oriented dendrites of principal neurons within the hippocampus (**K,N**, arrowheads) and cortex (**L,O**, arrowheads) along the dorso-ventral axis (highlighted in green). Note that the slightly distorted shape of the brain sections in the DWI image **(M)** is a consequence of the relatively long EPI readout of the measurement sequence. However, this only resulted in local geometrical shifts and does not invalidate the observed contrasts in diffusion measures. Scale bar: 2 mm (whole brain) and 500 μm (magnifications).

Analysis of a whole-brain section of a Thy1-eGFP mouse is shown in Figure 8. In addition to the features previously identified in hippocampal slices (Figures 8B,E,H,K,N), photomicrographs of the eGFP expression confirmed that both pyramidal and granule cells exhibit dendritic processes aligned in a very strict and parallel order, extending radially from their respective neuronal cell layer (Figure 8K, arrowheads). It should be noted that the whole-brain slices allowed to visualize other structural features in DWI, such as neocortical parallel streamlines oriented radially to the brain surface (Figure 8O, color-coded in green, arrowheads) matching the orientation of dendrites of principal neurons (Figure 8L).

**FIGURE 9:**
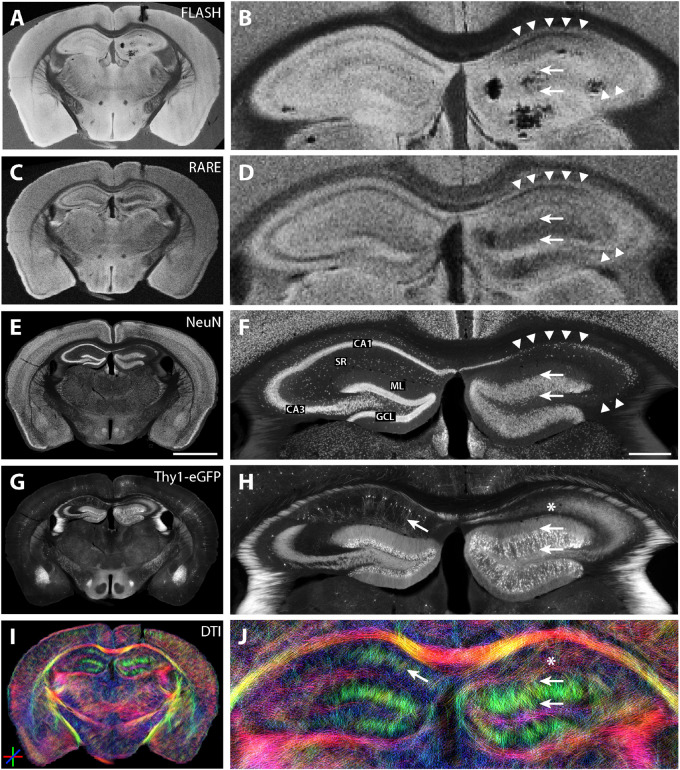
MR and histological images of a representative fixed whole-brain section from a chronically epileptic Thy1-eGFP mouse. **(A–D)** Structural MRI highlights morphological differences between the contralateral and the kainate-injected hippocampus **(B,D)**. While the granule cell layer thickness strongly increased on the ipsilateral side (arrows), contrast was reduced in the pyramidal cell layers CA1 and CA3 (arrowheads). Darker areas in **(B)** are susceptibility artifacts due to metal fragments from the injection needle after kainate injection – tissue is actually intact as seen in the corresponding immunohistochemical stainings (see **F,H**). NeuN staining **(E,F)** and eGFP expression **(G,H)** reveal the typical hallmarks of hippocampal sclerosis: dispersion of the GCL (arrows), loss of CA1 and CA3 pyramidal cells (arrowheads; CA, cornu ammonis; SR, stratum radiatum; GCL, granule cell layer; ML, molecular layer). **(I,J)** DWI shows elongated streamlines in the ipsilateral granule cell layer and molecular layer, most likely corresponding to granule cell dispersion, mossy fiber sprouting and gliosis (two arrows, see also Heinrich et al., 2006) for reference to astrogliosis after kainate injection) whereas dorso-ventral streamlines within areas of CA1 and CA3 pyramidal cell dendrites disappeared, reflecting the degeneration of these cells (asterisk). Other brain regions do not exhibit strong morphological changes after the KA treatment. Scale bars: 2 mm (whole brain: left column) and 500 μm (magnifications: right column).

One representative section from an epileptic mouse is shown in Figure 9. NeuN immunostaining and eGFP expression revealed the typical features of hippocampal sclerosis: dispersion of the GCL (Figures 9F,H, arrows) and the pronounced loss of CA1 and CA3 pyramidal cells (Figures 9F,H, arrowheads). As in the previous investigations in hippocampal slices, the increased thickness of the GCL and the neuronal cell loss in CA1 and CA3 pyramidal cell layers could be seen in structural MRI on the ipsilateral (kainate-injected) side (Figures 9B,D).

On the contralateral hippocampus, DWI showed dorso-ventral (DV) diffusion streamlines (color-coded in green) mainly in the molecular layer of the dentate gyrus and in the stratum radiatum of CA1. In the ipsilateral dentate gyrus, the streamlines appeared elongated (Figure 9J, two horizontal arrows), while in the region of CA1 stratum radiatum, DV streamlines could not be detected (Figure 9J, asterisks).

Ipsilateral and contralateral DWI features within the same slices could then be compared by paired *t*-tests. This confirmed previous results that FA of CA1 was significantly lower on the ipsilateral side (*p* = 0.01). Moreover, left–right diffusivity was significantly higher ipsilaterally, in accordance with the loss of anisotropy (*p* = 0.04). In the dentate gyrus, mean (*p* = 0.004), dorso-ventral (*p* = 0.009), and left–right (*p* = 0.004) diffusivities were all higher on the ipsilateral side, again in concordance with previous results (Figure 5), and thus demonstrating that DWI features could be used as a biomarker to identify the epileptogenic hippocampus in unilateral MTLE.

## Discussion

This study investigated high resolution MRI biomarkers of histological details of mouse brain tissue in epilepsy. The use of fixed samples greatly facilitated the validation of our acquired MR data by direct histological comparison of the same specimen, while circumventing issues of intra-scan variations such as physiological motion. Importantly, we made use of a MTLE mouse model in order to test the extent to which histopathological changes could be visualized by this method. While fixed tissue differs from living tissue in diffusion and relaxation parameters (Purea and Webb, 2006; Schmierer et al., 2008; Richardson et al., 2014; Birkl et al., 2016), the presented work still provides important clues on the cellular-level correlates of MR features that may be observed *in vivo* (Janz et al., 2017).

DWI of tissue sections from healthy control and kainate-injected, epileptic animals revealed distinct features arising from highly organized neuronal dendrites and axons. In detail, radially oriented neuronal dendrites with high density extending from CA1 and CA3 pyramidal cell layers and the granule cell layer of the dentate gyrus elicited strongly anisotropic water diffusion corresponding to the underlying neuronal morphology. In contrast to previous studies from, e.g., Zhang et al. (2002; Shepherd et al., 2007; Bohlen et al., 2014; Calabrese et al., 2015; Sierra et al., 2015), we could also show Schaffer collaterals as well as mossy fibers even in combination with tractography without any contrast agent. In particular, granule cell mossy fibers were seen as distinct DWI tracts, in accordance with a recent study demonstrating the ability of diffusion tractography to visualize hippocampal network organization (Wu and Zhang, 2016).

In the current work, we show that these features can be used for the detection of pathological alterations in epilepsy. The recent study of Stefanits et al. (2017) shows the importance of high-resolution imaging of hippocampal sclerosis for clinical aspects. In chronically epileptic animals, we found that histological features of the underlying neuronal network caused consistent and reproducible changes in the corresponding MR data. In detail, DWI was able to detect the degeneration of CA1 and CA3 pyramidal cells, dispersion of granule cells and the associated network rearrangement. Crucially, even more subtle modulations, such as the exclusive degeneration of CA1 pyramidal cells in the contralateral hippocampus, could be clearly detected by DWI as reduced fractional anisotropy in CA1 (Figures 4, 6). Overall, this study thus provided a better understanding of the cellular-level correlates of the diffusion alterations that manifest during epileptogenesis (Immonen et al., 2008; Sierra et al., 2015). Here, the validation of such features was made possible by using the exact same tissue samples for the MR experiments and histological examinations, given that pathological alterations are inherently variable across epileptic animals, and also show variations along the hippocampus within individual animals (Häussler et al., 2012).

Importantly, while DWI and histological correlations have been reported previously when pooling healthy and epileptic animals (Laitinen et al., 2010), here we also show that these correlations were also significant when considering only intra-group correlations within ipsilateral epileptic slices (Figure 7). Indeed, in addition to the specific structural differences associated with the pathology, localized damage due to the intrahippocampal kainate injection in the mouse model may drive common variance in the DWI and histological parameters, especially considering that control animals did not undergo sham injections. However, the observation of significant correlations within the epileptic group, in which all animals underwent the exact same experimental procedures, shows that the DWI features in CA1 and CA3 directly reflect the underlying cellular microstructure, i.e., the severity of the pathological changes, rather than being driven by other between-group differences. Interestingly, there was no significant intra-group correlation between GCL density and either MD or FA of the dentate gyrus. This suggests that changes in diffusion properties in the GCL are mainly driven by processes other than granule cell dendrites. Indeed, previous studies have reported the emergence of radial glial fibers in the GCL of epileptic animals, contributing to DWI metrics (Heinrich et al., 2006; Janz et al., 2017). While histological examinations remain the gold standard to study structural dynamics of neuronal networks in animal models, such longitudinal investigations require large numbers of animals in order to cover multiple time points (Bohlen et al., 2014; Richards et al., 2014; Suero-Abreu et al., 2014; Wu and Zhang, 2016). MRI offers the advantage of mapping cyto- and axonal architecture non-invasively (Calabrese et al., 2015), allowing longitudinal *in vivo* measurements in the same animals (Janz et al., 2017).

While it is clear that the spatial resolution of MRI cannot be expected to match that of histology, even when considering the ability of DWI to infer microstructural features occurring at the sub-voxel level, the identification of DWI biomarkers of neuropathological changes, particularly early in the course of epileptogenesis before first clinical manifestations, would be especially relevant. Our results show that diffusion parameters can be directly ascribed to microstructural features such as well-ordered neuronal cell layers and their respective cellular processes in pathologically altered tissue. It should however be noted that these findings are specific to the intrahippocampal kainate model and to MTLE. While this model has been extensively studied and mimics many of the histopathological and MR imaging features of human MTLE (Janz et al., 2017), it is clear that the modeling procedure notably involving an invasive intrahippocampal injection does not fully reflect the real situation in humans. Further studies will thus be required to determine to which extent the results are applicable to other types and models of epilepsy, and ultimately to human epilepsy patients. While investigating the relevance of DWI as an early biomarker in humans before the emergence of the first clinical symptoms would require extensive long-term prospective studies, a more accessible alternative would be the examination of resection samples in patients having undergone epilepsy surgery, which is also amenable to histological comparisons (Janz et al., 2017). This could provide first indications on the feasibility of detecting pathological microstructural alterations in human patients and could also guide the development of MR imaging protocols that would be ultimately applicable *in vivo*. Moreover, while the current study employed a simple mono-exponential diffusion model emphasizing typical features such as the orientation and amplitude of the diffusivity, more complex diffusion models have shown great potential in inferring even more details about the underlying microstructure of the imaged tissue (Bonilha et al., 2015; Gatto et al., 2019). An investigation of the benefits of this additional information would be well worth pursuing.

## Conclusion

We investigated fixed specimens of mouse brain tissue, which allowed the same tissue slices to be imaged using various MR protocols as well as subsequent fluorescence microscopy to achieve maximum comparability. The results show that high-resolution DWI parameters are associated with specific features of cellular architecture and neurodegeneration in MTLE.

The next step will be to monitor the described MR biomarkers in longitudinal studies to characterize the temporal evolution of these neuropathological cellular processes during epileptogenesis. In humans, *in vivo* measurements at sub-millimeter resolutions are gradually becoming more common (Yassa et al., 2010; Van Veluw et al., 2013; Chang et al., 2015) and a better understanding of the relation between MR signals and cellular-level microstructural effects will be of high relevance for clinical diagnostics.

## Data Availability Statement

The datasets generated for this study are available upon request to the corresponding author.

## Ethics Statement

The animal study was reviewed and approved by the regional council (Regierungspräsidium Freiburg) and local animal welfare officer, according to the German animal protection act.

## Author Contributions

KG-G, JG, RK, JH, JK, CH, and PL: conceived and designed the study. KG-G, JL, and DE: designed the MR protocols and performed MR data acquisition. KG-G and PL: MR data analysis. RK and JK: designed and manufactured hardware used in the study. JG and CH: performed animal handling, tissue preparation, and histological examination. KG-G, JG, CH, and PL: interpreted the data and wrote the manuscript. All authors read and approved the final manuscript.

## Conflict of Interest

The authors declare that the research was conducted in the absence of any commercial or financial relationships that could be construed as a potential conflict of interest.

## Footnotes

1 https://www.uniklinik-freiburg.de/mr-en/research-groups/diffperf/fibertools.html
