# Corrigendum: Histological Correlates of Diffusion-Weighted Magnetic Resonance Microscopy in a Mouse Model of Mesial Temporal Lobe Epilepsy

**DOI:** 10.3389/fnins.2020.00806

**Published:** 2020-08-19

**Authors:** Katharina Göbel-Guéniot, Johannes Gerlach, Robert Kamberger, Jochen Leupold, Dominik von Elverfeldt, Jürgen Hennig, Jan G. Korvink, Carola A. Haas, Pierre LeVan

**Affiliations:** ^1^Department of Radiology, Medical Physics, Medical Center – University of Freiburg, Freiburg, Germany; ^2^Faculty of Medicine, University of Freiburg, Freiburg, Germany; ^3^Experimental Epilepsy Research, Department of Neurosurgery, Medical Center – University of Freiburg, Freiburg, Germany; ^4^Department of Microsystems Engineering, Technical Faculty, University of Freiburg, Freiburg, Germany; ^5^BrainLinks-BrainTools Cluster of Excellence, University of Freiburg, Freiburg, Germany; ^6^Center for Basics in NeuroModulation (NeuroModulBasics), Faculty of Medicine, University of Freiburg, Freiburg, Germany; ^7^Institute of Microstructure Technology, Karlsruhe Institute of Technology, Karlsruhe, Germany; ^8^Department of Radiology and Department of Paediatrics, Cumming School of Medicine, University of Calgary, Calgary, AB, Canada; ^9^Hotchkiss Brain Institute and Alberta Children's Hospital Research Institute, University of Calgary, Calgary, AB, Canada

**Keywords:** MR microscopy, HARDI, tractography, hippocampus, mesial temporal lobe epilepsy, kainate

In the published article, there were errors in affiliations 2 and 3. Instead of “Experimental Epilepsy Research, Department of Neurosurgery, Medical Center – University of Freiburg, Freiburg, Germany” and “Department Neurosurgery, Experimental Epilepsy Research, Medical Center, University of Freiburg, Freiburg, Germany,” they should be “Faculty of Medicine, University of Freiburg, Freiburg, Germany” and “Experimental Epilepsy Research, Department of Neurosurgery, Medical Center – University of Freiburg, Freiburg, Germany,” respectively.

The authors apologize for these errors and state that this does not change the scientific conclusions of the article in any way. The original article has been updated.

