# Supplementary material for: Histological Correlates of Diffusion-Weighted Magnetic Resonance Microscopy in a Mouse Model of Mesial Temporal Lobe Epilepsy
Source: Front Neurosci. 2020 Jun 3;14:543. doi: 10.3389/fnins.2020.00543 (PMC7284165; doi:10.3389/fnins.2020.00543)
Supplement: Supplementary file 1 [file Table_1.DOCX]

| **MD [x10^-3^ mm²/s]** |  | **CA1** | **CA3** | **DG** | **hilus** | **srCA1** |
| --- | --- | --- | --- | --- | --- | --- |
|  | ipsi (mean ± std) | 0.47 ± 0.07 | 0.45 ± 0.08 | 0.51 ± 0.06 | 0.48 ± 0.08 | 0.41 ± 0.15 |
|  | contra (mean ± std) | 0.41 ± 0.13 | 0.44 ± 0.07 | 0.39 ± 0.04 | 0.38 ± 0.07 | 0.42 ± 0.15 |
|  | control (mean ± std) | 0.46 ± 0.05 | 0.48 ± 0.02 | 0.42 ± 0.06 | 0.45 ± 0.04 | 0.44 ± 0.02 |
|  | overall (mean ± std) | 0.45 ± 0.09 | 0.45 ± 0.07 | 0.45 ± 0.08 | 0.44 ± 0.08 | 0.42 ± 0.12 |
|  |  |  |  |  |  |  |
| **FA** |  | **CA1** | **CA3** | **DG** | **hilus** | **srCA1** |
|  | ipsi (mean ± std) | 0.18 ± 0.07 | 0.23 ± 0.05 | 0.31 ± 0.10 | 0.36 ± 0.07 | 0.30 ± 0.23 |
|  | contra (mean ± std) | 0.29 ± 0.12 | 0.28 ± 0.05 | 0.34 ± 0.09 | 0.36 ± 0.08 | 0.29 ± 0.11 |
|  | control (mean ± std) | 0.28 ± 0.03 | 0.26 ± 0.05 | 0.35 ± 0.12 | 0.26 ± 0.10 | 0.24 ± 0.09 |
|  | overall (mean ± std) | 0.24 ± 0.09 | 0.25 ± 0.05 | 0.33 ± 0.10 | 0.33 ± 0.09 | 0.29 ± 0.17 |
|  |  |  |  |  |  |  |
| **dvD [x10^-3^ mm²/s]** |  | **CA1** | **CA3** | **DG** | **hilus** | **srCA1** |
|  | ipsi (mean ± std) | 0.45 ± 0.07 | 0.45 ± 0.09 | 0.60 ± 0.11 | 0.39 ± 0.07 | 0.38 ± 0.16 |
|  | contra (mean ± std) | 0.39 ± 0.15 | 0.40 ± 0.08 | 0.46 ± 0.09 | 0.35 ± 0.09 | 0.36 ± 0.14 |
|  | control (mean ± std) | 0.52 ± 0.10 | 0.45 ± 0.09 | 0.49 ± 0.09 | 0.41 ± 0.09 | 0.41 ± 0.08 |
|  | overall (mean ± std) | 0.45 ± 0.11 | 0.43 ± 0.08 | 0.53 ± 0.12 | 0.38 ± 0.08 | 0.38 ± 0.13 |
|  |  |  |  |  |  |  |
| **lrD [x10^-3^ mm²/s]** |  | **CA1** | **CA3** | **DG** | **hilus** | **srCA1** |
|  | ipsi (mean ± std) | 0.45 ± 0.09 | 0.43 ± 0.08 | 0.43 ± 0.07 | 0.54 ± 0.16 | 0.40 ± 0.16 |
|  | contra (mean ± std) | 0.37 ± 0.16 | 0.42 ± 0.11 | 0.32 ± 0.08 | 0.35 ± 0.10 | 0.41 ± 0.18 |
|  | control (mean ±std ) | 0.39 ± 0.04 | 0.47 ± 0.03 | 0.36 ± 0.05 | 0.46 ± 0.04 | 0.44 ± 0.05 |
|  | overall (mean ± std) | 0.41 ± 0.11 | 0.44 ± 0.08 | 0.38 ± 0.08 | 0.46 ± 0.14 | 0.41 ± 0.14 |
|  |  |  |  |  |  |  |
| **dvlr_ratio** |  | **CA1** | **CA3** | **DG** | **hilus** | **srCA1** |
|  | ipsi (mean ± std) | 1.02 ± 0.13 | 1.05 ± 0.15 | 1.44 ± 0.35 | 0.77 ± 0.24 | 0.89 ± 0.32 |
|  | contra (mean ± std) | 1.12 ± 0.37 | 0.98 ± 0.20 | 1.54 ± 0.64 | 1.08 ± 0.44 | 0.92 ± 0.21 |
|  | control (mean ± std) | 1.35 ± 0.26 | 0.98 ± 0.24 | 1.36 ± 0.18 | 0.90 ± 0.15 | 0.98 ± 0.29 |
|  | overall (mean ± std) | 1.13 ± 0.27 | 1.01 ± 0.18 | 1.45 ± 0.41 | 0.90 ± 0.32 | 0.92 ± 0.27 |

Table S1. Mean values and standard deviations of the diffusion parameters for the hippocampal subregions CA1, CA3, DG, hilus and srCA1 within the different groups (ipsilateral, contralateral, control and overall).
